# Insufficient iodine intake in pregnant women in different regions of the world: a systematic review

**DOI:** 10.20945/2359-3997000000151

**Published:** 2019-07-12

**Authors:** Aline C. Candido, Núbia de S. de Morais, Luiza V. Dutra, Carina A. Pinto, Sylvia do C. C. Franceschini, Rita de Cássia G Alfenas

**Affiliations:** 1 Universidade Federal de Viçosa Departamento de Nutrição e Saúde Universidade Federal de Viçosa Viçosa MG Brasil Departamento de Nutrição e Saúde, Universidade Federal de Viçosa (UFV), Viçosa, MG, Brasil

**Keywords:** Iodine deficiency, pregnant woman, prevalence

## Abstract

**Objective:**

To determine the prevalence of insufficient iodine intake in pregnant women.

**Materials and methods:**

The search was performed in the electronic databases Medline (PubMed), Latin American and Caribbean Literature in Health Sciences (Lilacs) and Scopus. Review studies, experimental studies, those with adolescent pregnant women (< 20 years) and iodine supplementation were excluded. The selection followed the steps of identifying the articles in the databases, deleting the duplicates, and reading the titles, abstracts, and then the entire article. The search for the articles occurred in September 2017, using the descriptors “pregnant” and “iodine deficiency” NOT “supplementation” in English, Portuguese and Spanish.

**Results:**

Thirteen articles were included, the deficiency prevalence ranged from 16.1% to 84.0%, and the median of iodine intake was insufficient in 75% of the studies. There is no classification for mild, moderate or severe levels of iodine deficiency in pregnant women, which makes it impossible to know the real dimension of the problem.

**Conclusion:**

The high prevalence of insufficient iodine intake in pregnant women, observed worldwide, shows the need for a population classification in order to direct public policies. Arch Endocrinol Metab. 2019;63(3):306-11

## INTRODUCTION

Iodine is essential for the synthesis of thyroid hormones during pregnancy and for the fetal neurological development (1-3) The main consequences of low intake for pregnant women are goiter, spontaneous abortion, hypothyroidism and thyroid nodules. And for the fetus it can result in neonatal hypothyroidism, cretinism, retardation in growth and neuropsychomotor development (4).

In pregnant women, the recommendation for iodine is higher because there is an increase in the production of thyroid hormone, renal losses and transfer of this mineral from the mother to the fetus, all of which increases the need (5).

The availability of iodine in nature differs by geographical area and deficiency is more associated with mountainous regions such as the Himalayas and Alps and areas with frequent flooding. In addition, other regions also have a scarcity of this mineral, such as Central Africa, Central Asia, Europe and in places where the soils are poor (6).

Universal salt iodination was suggested in 1831 by the French scientist Boussingault to minimize the prevalence of goiter. As a result, this strategy reduced goiter in the population, increased urinary excretion, improved thyroid function and increased iodine intake in pregnant women, so it was implemented in several countries around the world (7,8).

Based on this information, the identification of iodine deficient countries allows us to build a global structure for the formulation of targeted and effective public policies. Therefore, our goal is to determine the prevalence of insufficient iodine intake in pregnant women.

## MATERIALS AND METHODS

The review followed the recommendations of the Preferred Reporting Items for Systematic Reviews (PRISMA) (9) and was based on the guiding question “Is there a reason for concern about insufficient iodine intake in pregnant women?”.

The article search occurred in September 2017 without date delimitation. The authors independently searched the electronic databases Publisher Medline (PubMed), Latin American and Caribbean Literature in Health Sciences (Lilacs), and Scopus. Descriptors indexed in the Health Science Descriptors system (Decs) were combined as follows: “pregnant” AND “iodine deficiency” NOT “supplementation”, in English, Portuguese and Spanish. For the PubMed search, we used the human, pregnant and adult filters, and in the Scopus filters, we used articles and pregnant women.

Original articles on the prevalence of insufficient iodine intake in adult pregnant women (≥ 20 years) based on the Urinary Iodine Concentration (UIC), according to data from World Health Organization (WHO), were included (10). Review studies, experimental studies, those with adolescent pregnant women (< 20 years old) and iodine supplementation were excluded. The selection followed the steps of identifying the articles in the databases, deleting the duplicates, and reading the titles, abstracts, and then the entire article.

The methodological quality of the studies was evaluated by the questionnaire proposed by Downs and Black (11), which contains 27 questions divided into four categories: study report (main findings described), external validity (evaluates representativeness), internal validity (investigates biases and confounding factors) and study power. We excluded 10 of the 27 questions since they referred to experimental studies. Each answer received a score of “0” (if it did not meet the criterion evaluated) or “1” (if the criterion was met), with a maximum of 17 points.

## RESULTS

**Figure 1:**
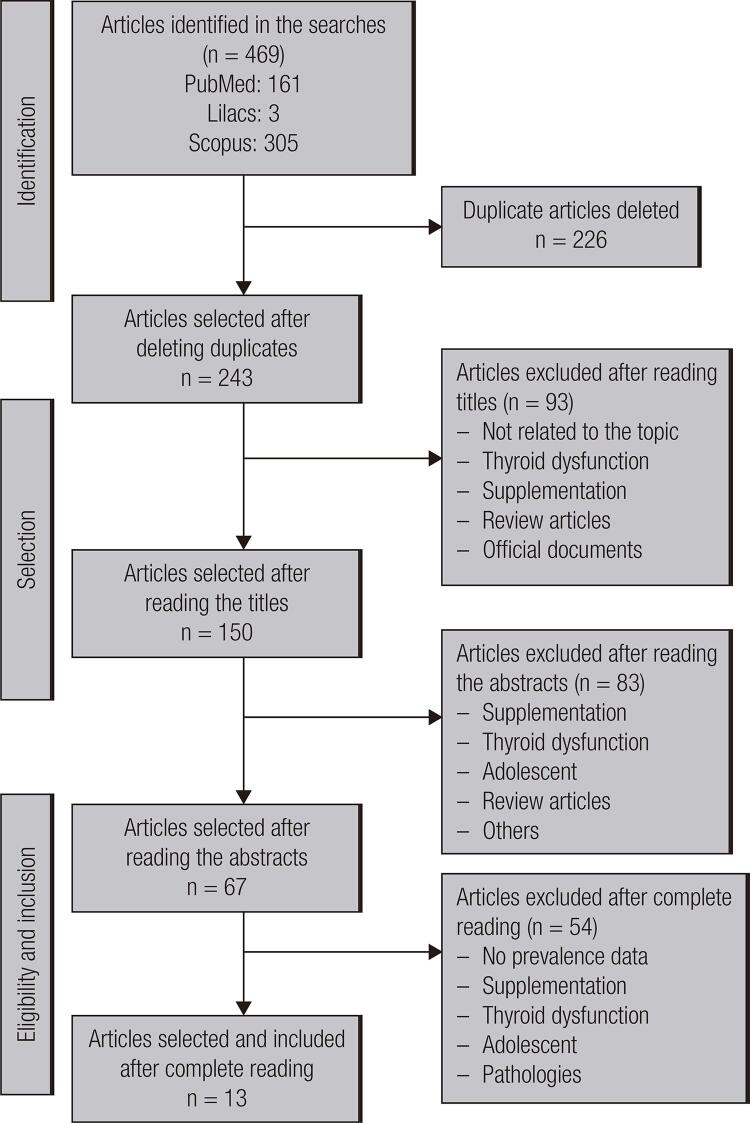
Flowchart of the process of identification and selection of the articles included. Source: PRISMA (9).

The search returned 469 articles. After eliminating duplicates by bases and among bases, 243 remained. After reading titles, abstracts and articles in full, 13 were included (Figure 1).

The years of the studies ranged from 2010 to 2016, with three longitudinal and 10 cross-sectional studies, performed in North and South America (Venezuela and Canada), Africa (Democratic Republic of Congo), Asia (Japan, Iran, Turkey, Bangladesh, India), Europe (England and Spain), and Oceania (Australia).

The sample size ranged from 36 to 5,256 pregnant women and the prevalence of insufficient iodine intake ranged from 16.1 to 84.0%. In order to evaluate the urinary iodine concentration (UIC) of pregnant women, the WHO reference was considered, where UIC < 150 μg/L is classified as insufficient iodine intake, 150-249 μg/L adequate, 250-499 μg/L more than necessary, ≥ 500 μg/L excessive intake (10).

Table 1 describes the results of the studies included in this review, with a median UIC ranging from 56.8 to 224.5 μg/L. As described in Table 1, we found that 75% (n = 9) of the studies were classified as insufficient iodine intake.

**Table 1:** Description of the studies selected for systematic review

Authors / Year	Site	Study Design	Recruitment	Sample size (n)	Trimester of pregnancy	Median Urinary Iodine Concentration (UIC) (μg/L)	Median UIC Classification	Prevalence of Iodine Deficiency	Quality of the Studies Included
Nguyen and cols. (2010) (12)	Australia	Cross-sectional	Pregnant women from any trimester of gestation seen at Canberra Hospital, Australia	100	The authors did not mention the trimester of gestation	62 (12-750)	Insufficient	84%	12 points
Caballero (2011) (13)	Venezuela	Cross-sectional	Pregnant women were selected at the prenatal visit. We selected 300 pregnant women, 100 women from each quarter	300	First trimester (n = 100), second trimester (n = 100) and third trimester (n = 100)	224.5	Adequate	25%	14 points
Fuse and cols. (2011) (14)	Japan	Cross-sectional	Healthy pregnant women were recruited during the 3 quarters and puerperal with 5 to 6 weeks postpartum without history of thyroid disease attended at Yamaguchi Hospital, Funabashi city	934	First trimester (n = 243), second trimester (n = 541), third trimester (n = 466) and postpartum (n = 533)	219	Adequate	16.1%	16 points
Çetinkaya and cols. (2012) (15)	Turkey	Cross-sectional	We recruited pregnant women in the three trimesters of gestation at the Medical University of Ataturk, Turkey	113	First trimester (n = 30), second trimester (n = 49) and third trimester (n = 34)	132.8 (15.1-291.6)	Insufficient	72.6%	9 points
Shamim and cols. (2012) (16)	Bangladesh	Cross-sectional	We recruit pregnant women from rural areas in northwestern Bangladesh. A randomized, placebo-controlled study with vitamin A or beta-carotene supplementation	2490	First trimester (≤ 16 weeks, n = 1376) and third trimester (≥ 32 weeks, n = 1114)	Initial: 66 (34 - 133) Final: 55 (28 -110)	Insufficient	Initial: 78.85¨% Final: 82.94%	16 points
Bath and cols. (2013) (17)	England	Longitudinal	Pregnant women living in the old Avon area in South West England.	1040	First trimester (< 13 weeks)	91.1 (53.8 - 143)	Insufficient	67%	13 points
Aguayo and cols. (2013) (18)	Spain	Longitudinal	Women attending obstetric outpatient appointments in the catchment area of Cruces Hospital	2104	First trimester (n = 2104) and second trimester (n = 1322)	Quarter 1st 88.5 (16 -875) 2nd 140 (21 – 880)	Insufficient	Quarter 1st – 79.8% 2nd – 54.4%	16 points
Amouzegar and Azizi (2013) (19)	Turkey	Cross-sectional	Pregnant women referred to the mother and child health care clinics of two maternity hospitals of Tehran	36	First trimester (< 15 weeks)	138.4 (24.1 – 404)	Insufficient	34.0%	16 points
Habimana and cols. (2013) (20)	Democratic Republic of Congo	Cross-sectional	375 pregnant women attending antenatal consultation, 125 women in each of the three maternity units	225	225 pregnant women in the three quarters, 75 women who gave birth and 75 non-pregnant women as controls	138 (105 – 172)	Insufficient	52.3%	13 points
Katz and cols. (2013) (4)	Canada	Cross-sectional	250 pregnant women from a clinical hospital	142	Second or third trimester of a singleton pregnancy	221 (142 – 397)	Adequate	29.6%	12 points
Joshi and cols. (2014) (21)	India	Cross-sectional	Pregnant women (n = 5256) attended the Jamnabai General Hospital.	5.256	First trimester (< 15 weeks)	297, 14	More than necessary	16.79%	10 points
Bath and cols. (2015) (22)	England	Longitudinal	230 British pregnant women recruited for the Selenium in Pregnancy intervention	230	Pregnant women in the first trimester (12-14 weeks), second trimester (20 weeks) and third trimester (35 weeks)	56.8 (31.1 – 104)	Insufficient	55.7%	15 points
Delshad and cols. (2016) (23)	Iran	Cross-sectional	Pregnant women attended at maternal and child health centers	1072	Singleton pregnancy and in the first, second and third trimester of their pregnancy	87.3 (43.5 – 139.1)	Insufficient	78.17%	10 points

In the evaluation of the methodological quality of the studies, the lowest score was nine and the highest 16. The best-evaluated criteria were the study report (main findings described), external validity (the same follow-up time for all individuals, appropriate statistical tests, and outcomes with reliable measures) and internal validity (individuals recruited in the same period). Only two studies presented power and adjustment for confounding factors in the analysis.

## DISCUSSION

Insufficient iodine intake is an obstacle to social and economic development, reaching approximately 2 billion people worldwide (10). To eradicate the disorders caused by deficiency, universal iodination of salt is used as a safe, economical and sustainable strategy to ensure adequate intake worldwide (24).

Globally, 86.6% of households have access to iodized salt, with the number of people consuming it increasing from 1 billion to approximately 4 billion in the last 10 years (25) In this study, of the evaluated countries, Japan has no legislation for salt iodination, in England and Spain, it is voluntary, and it is mandatory in the others.

The highest prevalence of deficiency detected was in Australia (84%), located in Oceania, different from expected, since there is a mandatory salt iodization policy and is surrounded by the Indian and Pacific oceans. However, natural disasters are recurrent in that country, and iodine deficiency can be an ecological phenomenon caused by flooding and soil erosion and, consequently, food crops will be deficient (4).

Asian countries: Turkey, Bangladesh and Iran showed insufficient iodine intake in pregnant women. Despite the extensive territory, the exponential growth of the population has led to a shortage of basic survival conditions, making access to adequate food difficult. This deficiency may also be a reflection of geographic characteristics such as mountains, floodplains and distance from the sea, restricting access to iodine sources.^10^ However, in India, iodine intake was more than necessary, demonstrating that monitoring salt iodization is critical, since overeating can be detrimental to health (21).

In contrast, the lower prevalence of deficiency was observed in Japan, which has no legislation for salt iodization. However, the habit of ingesting dietary sources of iodine, without subjecting them to high temperatures, may justify adequate population status (26).

Studies in European countries, England and Spain, found insufficient intake. The authors attributed this result to variations in the consumption of dietary sources of iodine and to the fact that pregnant women did not receive supplementation (18,22).

In Venezuela and Canada, located in South and North America, iodine intake was adequate, demonstrating the success of awareness campaigns conducted in these countries for the consumption of iodized salt to protect the mother from health problems (4,13).

At the 60th WHO World Health Assembly in 2007, about 31% of the world population had insufficient iodine intake and the most affected regions were Asia and Europe, while in the Americas the intake of fortified salt ensured adequate iodine status (25).

In Africa, a study in the Democratic Republic of Congo found insufficient iodine intake in the semi-urban and rural region, reflecting low socioeconomic status and population location, making access to iodine sources difficult. In addition, in the rural area, the habit of using natural spices instead of fortified salt contributes to this deficiency (20).

The difference in the prevalence of insufficient iodine intake can be attributed to geographic characteristics, dietary habits and salt iodination policy. This study was geographically representative allowing an overview of the prevalence of insufficient iodine intake in pregnant women in different regions of the world: the overall state of iodine in pregnant women.

The limitation is that the classification for levels of mild, moderate or severe iodine deficiency in pregnant women is not defined, making it impossible to know the magnitude of the problem.

In conclusion, pregnant women are a group vulnerable to insufficient iodine intake and the high prevalence observed in this review confirms the severity of this health problem worldwide. Therefore, there is a need for a population classification to guide public policies, as well as strategies such as salt iodization that should receive government support, and an effective monitoring to ensure adequate iodine intake.

## References

[1] Calvo RM, Jauniaux E, Gulbis B, Asunción M, Gervy C, Contempré B, et al. Fetal tissues are exposed to biologically relevant free thyroxine concentrations during early phases of development. J Clin Endocrinol Metab. 2002;87(4):1768-77. 10.1210/jcem.87.4.8434 11932315

[2] Morreale de Escobar G, Obregon MJ, Escobar del Rey F. Role of thyroid hormone during early brain development. Eur J Endocrinol [Internet]. 2004;(Suppl 3):U25-37. 10.1530/eje.0.151u025 15554884

[3] Teixeira D, Calhau C, Pestana D, Vicente L, Graça P. Iodo – Importância para a saúde e o papel da alimentação. Programa Nacional para a Promoção da Alimentação Saudável – Direção-Geral da Saúde; 2014.

[4] Katz PM, Leung AM, Braverman LE, Pearce EM, Tomlinson G, He X, et al. Iodine Nutrition During Pregnancy In Toronto, Canada. Endocr Pract. 2013;19(2):206-11. 10.4158/EP12193.OR PMC3968857 23186967

[5] Lazarus JH. Thyroid disease in relation to pregnancy: A decade of change. Clin Endocrinol (Oxf) [Internet]. 2000;53(3):265-78. 10.1046/j.1365-2265.2000.01087.x 10971442

[6] Linhares DPS, Garcia PV, Almada A, Ferreira T, Queiroz G, Cruz JV, et al. Iodine environmental availability and human intake in oceanic islands: Azores as a case-study. Sci Total Environ. 2015 Dec 15;538:531-8. 10.1016/j.scitotenv.2015.08.109 26318689

[7] Knobel M, Medeiros-Neto G. Moléstias associadas à carência crônica de iodo. Arq Bras Endocrinol Metabol. 2004;48(1):53-61. 10.1590/s0004-27302004000100007 15611818

[8] Pontes AAN De, Rocha ADM, Leite DFB, Lessa ADF, Adan LFF. Iodization of salt in Brazil, a controversial subject. Arq Bras Endocrinol Metabol. 2009;53(1):113-4. 10.1590/s0004-27302009000100017 19347194

[9] Liberati A, Altman DG, Tetzlaff J, Mulrow C, Gøtzsche PC, Ioannidis JPA, et al. The PRISMA statement for reporting systematic reviews and meta-analyses of studies that evaluate healthcare interventions: Explanation and elaboration. BMJ. 2009 Jul 21;339:b2700. 10.1136/bmj.b2700 PMC2714672 19622552

[10] WHO. Assessment of the iodine deficiency disorders and monitoring their elimination. World Heal Organ (WHO), Geneva [Internet]. 2007;3:1-107.

[11] Downs SH, Black N. The feasibility of creating a checklist for the assessment of the methodological quality both of randomised and non-randomised studies of health care interventions. J Epidemiol Community Health. 1998;52(6):377-84. 10.1136/jech.52.6.377 PMC1756728 9764259

[12] Nguyen B, Baker D, Southcott E, Potter J, Sneddon A, Hickman PE. Iodine deficiency in pregnant women in the ACT. Aust New Zeal J Obstet Gynaecol. 2010;50(6):539-42.

[13] Caballero L. Yoduria en escolares y embarazadas del estado Trujillo, Venezuela 2007-2008 Urinary Iodine in school children and pregnant women of Trujillo state, Venezuela 2007-2008. Raem No. 2011;48(4):206-11.

[14] Fuse Y, Ohashi T, Yamaguchi S, Yamaguchi M, Shishiba Y, Irie M. Iodine status of pregnant and postpartum Japanese women: Effect of iodine intake on maternal and neonatal thyroid function in an iodine-sufficient area. J Clin Endocrinol Metab. 2011;96(12):3846-54. 10.1210/jc.2011-2180 21956426

[15] Çetinkaya K, Ingeç M, Çetinkaya S, Kaplan I. Iodine defi ciency in pregnancy and in women of reproductive age in Erzurum, Turkey. 2012;42(4):675-80.

[16] Shamim A, Parul C, Schulze K, Ali H, Alamgir K, Rashid M, et al. Iodine status in pregnancy and household salt iodine content in rural Bangladesh. Matern Child Nutr. 2012;8(2):162-73. 10.1111/j.1740-8709.2010.00282.x PMC6860684 20977661

[17] Bath SC, Steer CD, Golding J, Emmett P, Rayman MP. Effect of inadequate iodine status in UK pregnant women on cognitive outcomes in their children: Results from the Avon Longitudinal Study of Parents and Children (ALSPAC). Lancet [Internet]. 2013;382(9889):331-7. 10.1016/S0140-6736(13)60436-5 23706508

[18] Aguayo A, Grau G, Vela A, Aniel-Quiroga A, Espada M, Martul P, et al. Urinary iodine and thyroid function in a population of healthy pregnant women in the North of Spain. J Trace Elem Med Biol. 2013;27(4):302-6. 10.1016/j.jtemb.2013.07.002 23992867

[19] Amouzegar A, Azizi F. Variations of urinary iodine during the first trimester of pregnancy in an iodine-replete area. Comparison with non-pregnant women. Hormones. 2013;12(1):111-8. 10.1007/BF03401292 23624137

[20] Habimana L, Twite KE, Wallemacq P, De Nayer P, Daumerie C, Donnen P, et al. Iodine and iron status of pregnant women in Lubumbashi, Democratic Republic of Congo. Public Health Nutr. 2013;16(8):1362-70. 10.1017/S1368980012005484 PMC10271445 23324455

[21] Joshi K, Nair S, Khade C, Rajan MGR. Early gestation screening of pregnant women for iodine deficiency disorders and iron deficiency in urban centre in Vadodara, Gujarat, India. J Dev Orig Health Dis [Internet]. 2014;5(01):63-8. 10.1017/S2040174413000470 24847692

[22] Bath SC, Furmidge-Owen VL, Redman CW, Rayman MP. Gestational changes in iodine status in a cohort study of pregnant women from the United Kingdom: season as an effect modifier. Am J Clin Nutr. 2015;101(6):1180-7. 10.3945/ajcn.114.105536 PMC4441812 25948667

[23] Delshad H, Touhidi M, Abdollahi Z, Hedayati M, Salehi F, Azizi F. Inadequate iodine nutrition of pregnant women in an area of iodine sufficiency. J Endocrinol Invest. 2016;39(7):755-62. 10.1007/s40618-016-0438-4 26951055

[24] World Health Organization (WHO). World Summit for Children – Mid-Decade Goal: Iodine Deficiency Disorders (Idd). UNICEF-WHO Jt Comm Heal Policy Spec Sess. 1994;2(27):27-8.

[25] Pretell E. Boletim de Carências Nutricionais: Distúrbios por Deficiência de Iodo – DDI. Ministério da Saúde. Secretaria de Atenção à Saúde Política de Alimentação e Nutrição (CGPAN). 2008; 1: 1-6.

[26] Fisher J, Tran T, Biggs B, Tran T, Dwyer T, Casey G, et al. Iodine status in late pregnancy and psychosocial determinants of iodized salt use in rural northern Viet Nam. Bull World Health Organ. 2011;89(11):813-20. 10.2471/BLT.11.089763 PMC3209728 22084527

